# Chemerin contributes to *in vivo* adipogenesis in a location-specific manner

**DOI:** 10.1371/journal.pone.0229251

**Published:** 2020-02-24

**Authors:** David J. Ferland, Hannah Garver, G. Andres Contreras, Gregory D. Fink, Stephanie W. Watts

**Affiliations:** 1 Department of Pharmacology and Toxicology, Michigan State University, East Lansing, Michigan, United States of America; 2 Department of Large Animal Clinical Sciences, Michigan State University, East Lansing, Michigan, United States of America; State University of Rio de Janeiro, BRAZIL

## Abstract

Since chemerin’s identification as an adipokine, it has been associated with a number of human diseases including diabetes and obesity. However, the basic scientific foundation for these clinical determinations is still lacking. Fibroblastic mouse 3T3 cells are unable to develop lipid droplets if chemerin is not present. Thus, we hypothesized that an *in vivo* rat model chemerin knockout (KO; an advancement from the previously mentioned *in vitro* cultures) would have limited accumulation of lipid in adipocytes compared to their wild-type (WT) counterparts. Female WT/KO rats (Sprague Dawley background) were fed a low-fat diet starting at 8 weeks of age with weekly body weight and food consumption monitoring. At 25 weeks of age, adipose tissue depots were dissected and flash frozen for PCR analysis or fixed with paraformaldehyde for histology. Over the 17 weeks of experimentation, WT and KO animals did not have differences in total body weight or food consumption but KO animals had a significantly reduced amount of visceral fat compared to WT animals (via microCT at 8 and 25 weeks). Histology of retroperitoneal and mesenteric depots demonstrated a significant leftward shift in adipocyte size in the mesenteric but not the retroperitoneal depot of the KO compared to WT animals. Similarly, in the mesenteric fat of the KO rat, gene expression of adiponectin, fatty acid synthase, perilipin, and leptin were significantly reduced compared to mesenteric fat of WT animals and retroperitoneal fat of both WT and KO animals. Adiponectin was highlighted by a protein-protein interaction network as being important for the physiological effects of chemerin removal. These data are the first, to our knowledge, to demonstrate chemerin’s adipokine potential *in vivo* and identify it as fat depot location-specific.

## Introduction

With the rising obesity crisis around the world, obesity-related diseases such as diabetes [1], non-alcoholic fatty liver disease [2], high blood pressure [3], and even a compound pathology like metabolic syndrome [4] are now more frequent. Metabolic syndrome is defined as having three of the following five characteristics: high blood pressure, high BMI, dyslipidemia, impaired insulin sensitivity, and a large waist circumference [5]. To better treat patients and predict their prognosis, physicians have looked for blood markers of each of these diseases. Circulating concentrations of adiponectin (low) and leptin (high) are often thought of as classic markers of obesity-related diseases. However, a new class of adipokines, including vaspin and chemerin, also have strong positive correlations to these same diseases [6,7].

Chemerin plasma levels increase in diabetes [8,9], increased visceral adiposity [10,11], dyslipidemia [12,13], and high blood pressure [14,15] independently but these positive correlations often become even more powerful in people diagnosed with metabolic syndrome [16–18]. Chemerin is made in various tissues of the body but is most prominently produced by the liver and white adipose tissue [17]. Clinicians and basic scientists have implicated the abdominal visceral white adipose tissue as the major contributor of chemerin from fat [10,17] and that chemerin production is substantially lower in the brown adipose tissue compared to the white adipose tissue. After secretion, the binding of chemerin with chemerin receptor 1 initiates a number of different actions depending on the cell type it is affecting: chemotaxis on immune cells [19], calcium flux in smooth muscle cells [20], or matrix metalloproteinase activity for vascular growth [21].

Basic science research on chemerin suggests a link to inflammation: specifically how various forms of chemerin may [19] or may not [22] induce migration of immune cells. In 2007, several papers using shRNA knockdown of chemerin in the 3T3-L1 mouse fibroblast cell line–a classic adipogenesis model that can differentiated into adipocytes–demonstrated that chemerin and its receptor were necessary for lipogenesis or accumulation of lipid [17, 23]. Adipogenesis as a whole is likely regulated through chemerin’s influence over cyclins A2 and B2 [24].

This current study focuses on the impact of a germline chemerin knockout on adipogenesis and lipogenesis. We hypothesized that the chemerin knockout rat, when compared to its WT counterpart, would have impaired white adipose tissue lipid accumulation and reduced expression of genetic markers of adipogenesis and lipogenesis related to lipid accumulation like PPAR gamma, adiponectin, perilipin, fatty acid synthase, and leptin [reflecting what was previously seen with 3T3-L1 cells [23]].

## Materials and methods

### Animal care and handling

All procedures that involved animals were performed in accordance with the Institutional Animal Care and use Committee (AMEND201900134 / PROTO201900089) of Michigan State University that approved this study. Animals were maintained on a 12/12 hour light/dark cycle at a temperature of 22–25°C.

### Animal model

The knockout of the chemerin gene in the Sprague Dawley rat has been previously validated [25] and all animals were genotyped by ear punch at weaning. Female KO and WT rats were fed a normal chow (Envigo Teklad 22/5 #8640; 17% kcal from fat) from weaning until 8 weeks of age then switched to a low-fat normal diet (Research Diets D12450J; 10% kcal from fat) from 8 weeks till 25 weeks of age. Total body weight and food consumption were measured weekly.

### MicroCT

MicroCT scans of rats were performed by a core facility at Michigan State University using the Perkin Elmer Quantum GX microCT Imaging System (Waltham, MA, USA). The following image acquisition scan parameters were standardized and used at each scan interval time point: scan mode, Standard; gantry rotation time, 2-minutes; power, 90kVp/88uA; Field of View (FOV), 72mm; number of slices, 512; slice thickness, 144um; voxel resolution, 144um^3^. Animals were maintained on isoflurane (1–3%) and given supplemental heat while monitoring respiratory rate and heart rate. Image rendering, tissue segmentation, and analysis were performed using Caliper AnalyzeDirect^©^ (v12.0, Biomedical Imaging Resource, Mayo Clinic, Rochester, MN). Transverse images were taken at the same abdominal point on each animal. Instances where the field of view was too small to adequately measure subcutaneous fat, resulting in a Hounsfield unit standard deviation above 70, were excluded from the study due to an unreliable threshold analysis.

### Tissue collection

Animals were euthanized at 25 weeks of age with 2% isoflurane followed by pneumothorax. Liver, heart, kidney, ovarian/uterine fat, and retroperitoneal fat were removed in their entirety and weighed. Samples of retroperitoneal fat and mesenteric fat were fixed with 4% paraformaldehyde for histology or flash frozen in liquid nitrogen for mRNA analysis. Samples placed in paraformaldehyde were transferred to 30% ethanol after 24 hours before sending to the MSU Histopathology lab for sectioning and staining.

### Western blot

Blood was centrifuged at 500 *xg* for 20 minutes at 4°C. Plasma was diluted 1:25 and a bicinchoninic acid assay was used to determine protein content (#BCA1, Sigma Chemical Co, St. Louis, MO, USA). Fifty micrograms of protein was loaded into a 15% polyacrylamide gel run at 120 V. Protein was transferred for 1 hour at 100 V to a PVDF-FL membrane (#IPFL00010, EMD Millipore, Billerica, MA, USA). After drying, Total protein stain was added, imaged, and reverted (#926–11011, Li-Cor). Samples were blocked with chick egg ovalbumin for 3 hours. Chemerin primary antibody (1:1000; Abcam Cat# ab112520, Cambridge, MA, USA; ab registry: AB_10864055) was incubated with the membrane for 48 hours at 4°C, washed three times, and secondary antibody (1:1000; IRDye 800 anti-Mouse, LI-COR Biosciences Cat# 926–32210, Lincoln, NE, USA; ab registry: AB_621842) was incubated for one hour at 4°C. With total protein as a loading control, blots were visualized with the Odyssey CLx Infrared Imaging system (Li-Cor) and quantified with Image Studio (5.2.5, LI-COR Image Studio Software). When using Image Studio to perform the analysis, modification of LUTs does not affect the signal quantification.

### Histology and adipocyte analysis

Retroperitoneal and mesenteric fat samples were stained with H&E and imaged with a Nikon (Tokyo, Japan) Eclipse Ti microscope using a Nikon DS-Fi3 camera. Five fields of each biological replicate were collected at 4x magnification for quantification. Uncompressed tif files were analyzed for adipocyte area by the Adiposoft plugin (v. 1.15) for ImageJ Fiji (v 2.0.0). The Adiposoft plugin gated for adipocytes with an equalized diameter between 25 and 200 microns used a “micron per pixel” ratio of 0.86 according to the calibration of the microscope. Images were spot checked for appropriate quantification. Data represent a biological replicate n = 5 for each condition. All image capture and analysis were carried out with random identifiers to blind the operator to the genotype of the animal.

### PCR

Flash frozen tissue was processed by the Zymo Research (Irvine, CA, USA) QuickRNA kit for RNA isolation. Reverse transcription was performed by the Applied Biosystems (Foster City, CA, USA) High Capacity cDNA Reverse Transcription kit and the Applied Biosystems SimpliAmp Thermal Cycler at the following conditions: 10 minutes 25°C, 2 hours at 37°C, 5 minutes at 85°C, then hold at 4°C. PCR was performed with Applied Biosystems Quant Studio 7 Flex and Applied Biosystems Fast SYBR Green Master Mix under the following conditions: 95°C 20 sec, 40x 95°C 1 sec then 60°C 20 sec, then melt using 95°C 15 sec and 60°C 1 min. Sigma Aldrich (St Louis, MO, USA) KiCqStart SYBR Green primer sequences can be found in S1 Table. Beta-2-microglublin was used as a reference gene.

### Cytoscape network

PCR ΔC_T_ values from KO mesenteric adipocytes were divided by WT counterparts for a fold change. Genes that did not have statistically significant differences when comparing the ΔC_T_ means of KO and WT adipocytes were assigned a fold change of one. Protein interactions from the tested genes were imported to a Cytoscape (3.7.1) network using the STRING app (1.4.2) and STRING database (string-db.org) which compiles known protein-protein interactions from the literature in specific species of animal. All analyses were performed using the rat database. These interactions were mapped to edge width. Fold changes from experimental data were mapped to node color.

### Statistics and analysis

All samples, images, and data were coded so that all handling and initial analyses were blinded before the final statistical analysis. Statistics and graphing were performed with Prism (v.8, Graphpad). Time course data was analyzed with a 2-way ANOVA. Analyses comparing the WT and KO animals were performed with a two-tailed students t-test. PCR data comparing the gene expression of four groups utilized a one-way ANOVA with Sidak correction. Significance was considered p < 0.05. Histograms were analyzed for significant shifts with the Kolmogorov-Smirnov test for distributions. PCR data is represented as 2^-ΔC^_T_ where ΔC_T_ is the difference between the target gene and the beta-2 microglobulin reference gene. All genes were run with a technical duplicate which was averaged for subsequent analysis. All data in figures represent mean ± SEM based on the biological replicates (n).

## Results

### KO rats have reduced visceral adiposity

**Fig 1:**
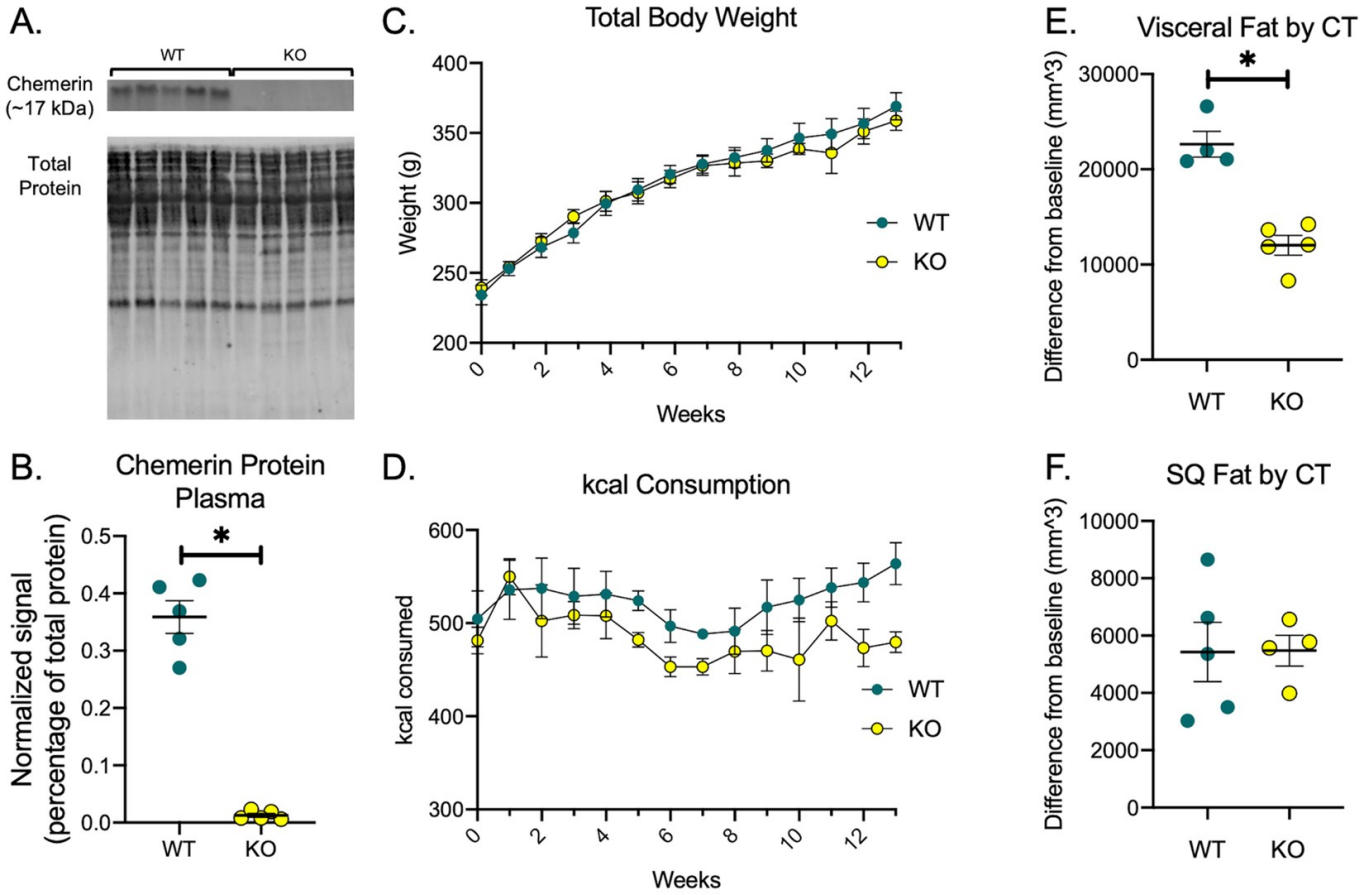
KO rats have reduced visceral adiposity. In addition to genotyping done at weaning, chemerin knockout was confirmed by loss of chemerin protein in plasma as measured by Western blot (A-B). Body weight of the rats were measured weekly (C) and kcal consumption was calculated based on food weight consumed multiplied by kcal density (D). Visceral fat (E) and subcutaneous (SQ) fat (F) was calculated by taking the difference between the 8 week and 25 week measurements. Only visceral fat was changed by genotype. All points and bars represent mean ± SEM for a biological replicate N = 5. * represents p < 0.05 for the respective statistical test. In the WT group of panel E and KO group of panel F, one animal was excluded due to Hounsfield unit variability discussed in the methods section. Time course data was analyzed by 2-way ANOVA and comparison of two groups was analyzed by two-tailed students t-test.

Rats were genotyped at weaning but chemerin knockout was confirmed by testing for chemerin protein in the plasma of WT and KO rats (Fig 1A and 1B). Over the course of 17 weeks, female WT and KO rats did not have different total body weights or kcal consumption (Fig 1C and 1D). MicroCT performed at 8 and 25 weeks of age suggests that while the adiposity of both the visceral and subcutaneous fat beds increased in all animals over time, accumulation of visceral but not subcutaneous fat in the KO rat was blunted (Fig 1E and 1F).

### Chemerin’s adipogenic potential is location-specific

**Fig 2:**
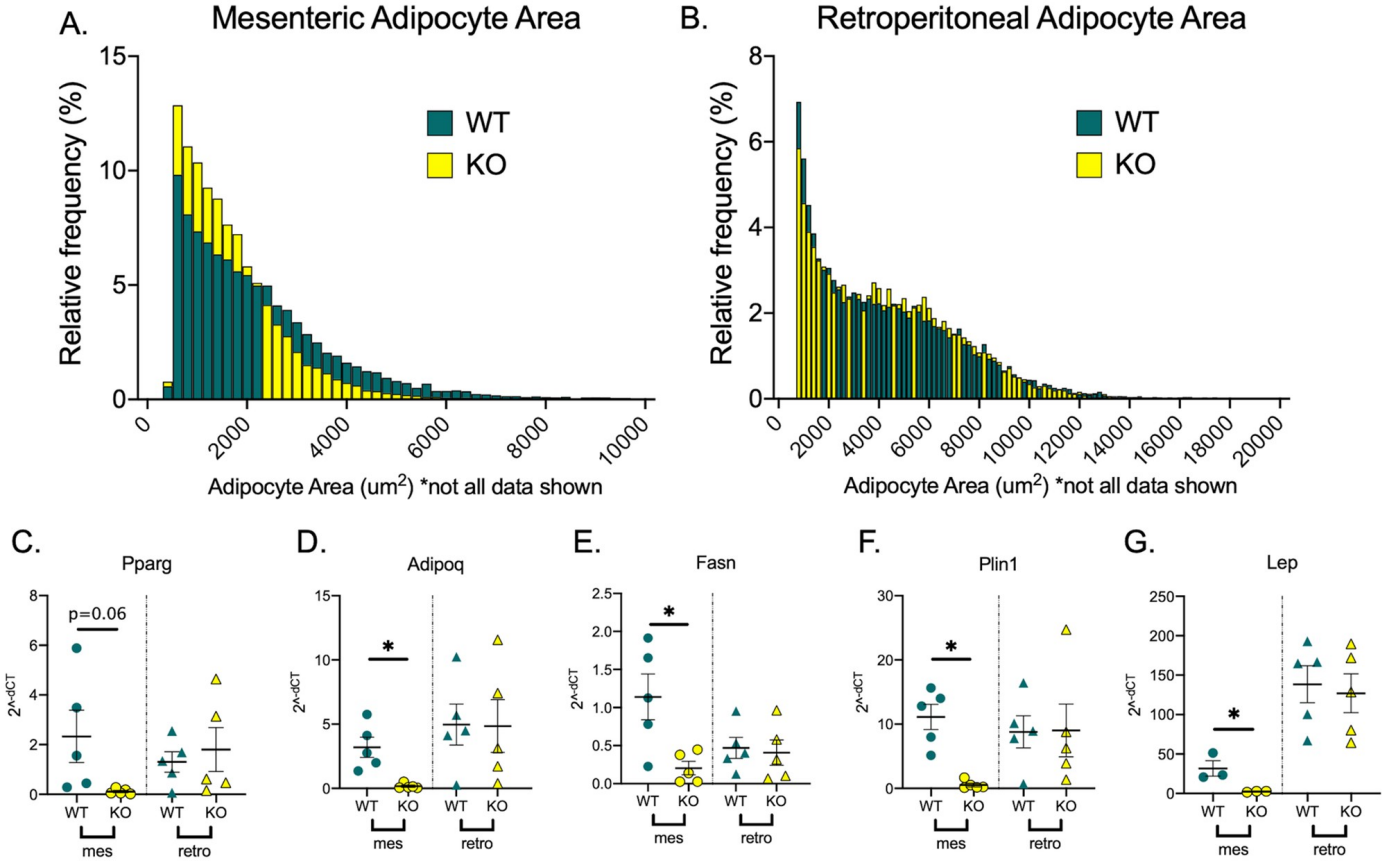
Chemerin’s adipogenic potential is location-specific. Histograms of adipocyte area show that mesenteric adipocytes with chemerin knocked-out have reduced area (A) whereas in retroperitoneal fat, there is no difference between WT and KO (B). Statistical significance of shifts in the histogram were performed by Kolmogorov-Smirnov test of frequency distribution with a p < 0.05. Five images of each biological replicate were analyzed and combined to create the histogram. Each bin was normalized to a percent of the total count for that individual tissue. To see distribution changes early on the x-axis, the x-axis was shortened and does not include some data at higher adipocyte sizes. PCR analysis showed that in these KO mesenteric adipocytes, adiponectin (*adipoq*), fatty acid synthase (*fasn*), perilipin (*plin1*), and leptin (*lep*) were significantly reduced. PPAR gamma (*pparg*) did not show any statistical significance (C-G). All points and bars represent mean ± SEM for a biological replicate N = 5. * represents p < 0.05 for the respective statistical test.

The two major components of visceral fat measured by CT were mesenteric and retroperitoneal fat. When adipose tissue was fixed, imaged, and analyzed for adipocyte area, the mesenteric adipocytes of the KO animals were significantly smaller than the WT animals (leftward shift in the histogram statistically confirmed by the Kolmogorov-Smirnov test for distributions; Fig 2A). By contrast, the adipocyte sizes from the retroperitoneal fat were not different between WT and KO animals (Fig 2B).

The mesenteric and retroperitoneal fats from WT and KO animals were tested for mRNA expression of genes related to adipocyte development. There was no statistical significance (p = 0.6) in the expression of *PPAR gamma* but we did observe reduced levels of adiponectin (*adipoq*), fatty acid synthase (*fasn*), perilipin (*plin1*) and leptin (*lep*; Fig 2C–2G) in the mesenteric but not retroperitoneal fat of the KO rat. This trend did not exist in the WT animals.

### Adiponectin is important to the actions of chemerin

**Fig 3:**
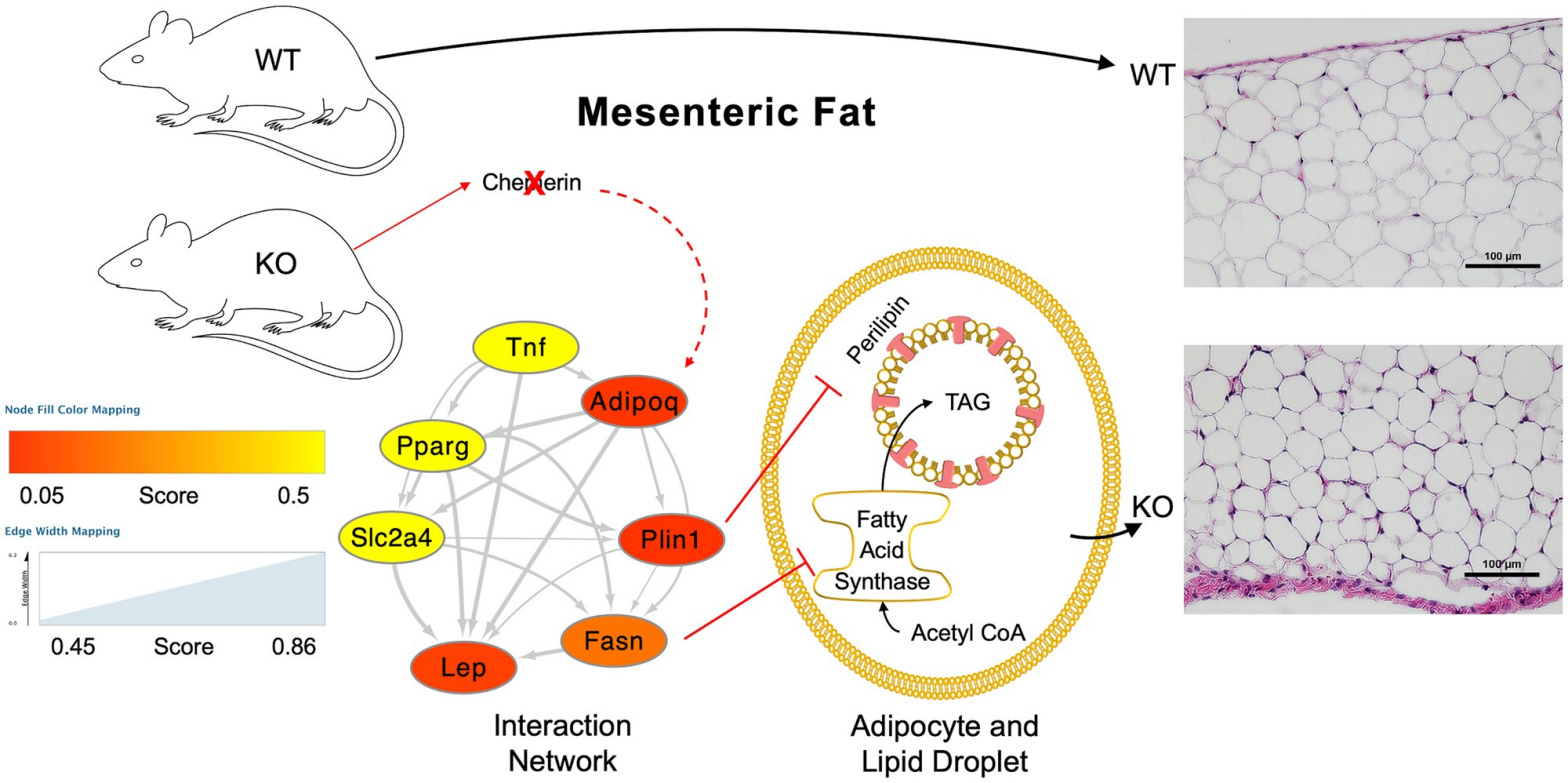
Adipocyte development network in chemerin KO rats. This network represents protein-protein interactions curated by the STRING database (edges with arrows) and gene expression data from this study (nodes). Edges are mapped according to the score given by the STRING app (v1.4.2) database correlating to the strength of data supporting their directional interaction. Scores for node mapping were determined by taking the 2^-ΔC^_T_ from chemerin KO mesenteric adipocytes and dividing them by the 2^-ΔC^_T_ from their WT counterparts. Comparisons of gene expression from KO and WT fats in Fig 2 that were not statistically significant were given a score of 1. Genes that were not incorporated into the STRING network (ccng2, ccna2, and tcf21) were not included in this figure. When expression is decreased with chemerin KO versus WT, the node will appear more red. When expression is unchanged, the node will appear yellow. These data were incorporated into the known physiology of the adipocyte and representative images of the WT and KO mesenteric adipose tissue to provide a hypothesis of the system discussed in this manuscript. Scale bars represent 100 um.

When gene expression data gathered from this study was overlaid on a directional protein-protein network of the genes tested in the mesenteric adipocytes of WT and KO, adiponectin was determined to be an important upstream gene affected by the removal of chemerin (Fig 3). The color of the nodes in the network represent the gene expression changes (Fig 2) while the width of the edges (with arrows) represent literature data curated by the STRING app within Cytoscape [26].

## Discussion

### Chemerin is a regulator of adipocyte development in mesenteric fat

Given the rapid expansion of epidemiological references to chemerin in the human literature, there is a dire need for basic science to substantiate the foundations of chemerin’s associations with different pathologies like obesity, blood pressure, and inflammation. Through a novel chemerin KO rat, this study provides important *in vivo* proof that chemerin is a contributor to the adipogenesis in a site-specific way.

From a basic science perspective, the mechanisms by which distinct chemerin isoforms contribute to adipocyte pathophysiology are still a mystery. Cleavage of a single amino acid from the C-terminus of the chemerin protein can dramatically alter affinity and efficacy for the chemerin receptor 1 and thereby alter the chemotactic rates of immune cells [19,27,28]. It is probable that the chemerin isoform milieu of the adipose tissue determines how chemerin supports a healthy or pathogenic environment. This study confirmed that chemerin production in the whole, healthy animal does influence the adipocyte development and supports the need for future research investigating how the actions of chemerin on the adipocyte change in metabolic syndrome.

An unexpected finding with important pathophysiological relevance is the location-specific changes in adipocyte-development with chemerin KO. Visceral adiposity has long been associated with negative outcomes in obesity and high blood pressure [29,30]. Researchers have slightly different definitions of visceral fat in rodents versus humans, which makes the integration of clinical and basic science data somewhat difficult. In rodents, visceral fat is defined based on vascular drainage (fat whose blood drains to the hepatic portal vein; mesenteric and omental fat but not retroperitoneal fat) while clinically it is based on what can be differentiated from subcutaneous adipose on a CT scan (mesenteric, omental, and retroperitoneal fat) [31].

Omental fat does not exist in the rat so it can be excluded from the discussion [31]. But it is also important to note that mesenteric fat is mostly perivascular to the mesenteric resistance vessels, which have substantial exposure to nutrients coming from the intestine which could exert significant control over total peripheral resistance [32,33]. With mesenteric adipose tissue better poised to influence the development of metabolic syndrome, it is not surprising that of the two adipose depots, chemerin would play a larger role in the adipocyte development of the mesenteric, rather than the retroperitoneal, fat.

### Interactions of chemerin with other regulators of the adipocyte

The genes that showed reduced expression in the mesenteric KO fat (adiponectin, perilipin, fatty acid synthase, and leptin) may adequately explain the reduction in size of the adipocytes. While the direct actions of adiponectin are catabolic, adiponectin is widely recognized as an adipokine that promotes healthy function. It ensures that adequate lipid is available for adipocyte storage and limits ectopic lipid accumulation [34]. Perilipin surrounds the lipid droplet and is responsible for maintaining the droplet in the adipocyte [35]. Fatty acid synthase is a key enzyme in the packing of lipid into the lipid droplet of the adipocyte [36]. Leptin is considered an endocrine signal of the amount of adipose tissue [34]. The reduced adipose tissue levels in the mesenteric fat have likely led to the reduced leptin expression in our PCR measurements. We tested other genes that might be critical to the preadipocyte development like *tcf21* or cell cycle regulators like *ccna2* or *ccng2*, but none of these were significantly changed in the mesenteric fat of the KO rat. From these data, it is possible that the adipogenesis rate was not affected and chemerin only influenced the adipocyte size. We expected PPAR gamma to also be reduced based on previous studies with chemerin in 3T3 cells [23]. While our data on PPAR gamma were inconclusive, it is likely that PPAR gamma activation is upstream of chemerin activation. One report proposed that the chemerin promoter contains a PPAR gamma binding motif [24]. If PPAR gamma expression is upstream of the chemerin gene we knocked out, that could possibly account for the lack of change in PPAR gamma expression.

The network of protein interactions combined with gene expression data outlines one possible way chemerin could influence the actual accumulation of lipid in these mesenteric adipocytes. Even without the involvement of PPAR gamma, chemerin can act at the level of adiponectin to influence the lipid in the cells. One paper describing the effects of chemerin, the chemerin receptor 1, and adiponectin in bovine adipocytes found that both adiponectin and chemerin itself cause expression of the chemerin gene [37]. These data combined with our protein network analysis imply a positive feedback loop where chemerin and adiponectin are able to promote adipocyte growth and lipid expansion. This is congruent with other reports where chemerin expression increases throughout differentiation [17,23,37–39].

## Limitations

Though the scope of this current study was on the interactions between chemerin and fat metabolism, it is important to remember that fat is not the only source of chemerin in the body. We did not study chemerin expression in the liver or adrenal–two other major sources of chemerin. While it is possible that chemerin from other sources could influence adipocyte development, the changes in lipid size were location dependent. This indicated a local effect of chemerin on metabolism of the adipocyte rather than a systemic one.

This study utilized female rats and while our previous validation of this transgenic model identified some minor difference in blood pressure regulation between males and females, most other parameters, including clinical blood chemistries, indicated a similar metabolic function [25]. As such, we would expect experiments in male rats to exhibit similar proportional changes between KO and WT animals.

## Conclusions

Chemerin may not play a global role in adipogenesis, as evidenced by the lack of changes in the total body weight and retroperitoneal adipocyte size in our chemerin KO rats. Yet, through these data, we show that chemerin plays an important *in vivo* role in the mesenteric fat, a fat depot that plays an important role in the pathogenesis of metabolic syndrome. This study provides a foundational understanding for current and future epidemiological research but also serves as a first step for future *in vivo* basic science chemerin research. We have demonstrated for the first time that chemerin plays a supportive role in adipocyte development of the rat but this effect is specific to the mesenteric adipose depot.

## Supporting information

S1 Table Forward and reverse sequences for Sigma-Aldrich KiCqStart SYBR primers used in this study. (TIF)

S1 Original western (TIF)

S2 Original western (TIF)
