# Supplementary figures and images for: Chemerin contributes to in vivo adipogenesis in a location-specific manner
Source: PLoS One. 2020 Feb 24;15(2):e0229251. doi: 10.1371/journal.pone.0229251 (PMC7039425; doi:10.1371/journal.pone.0229251)

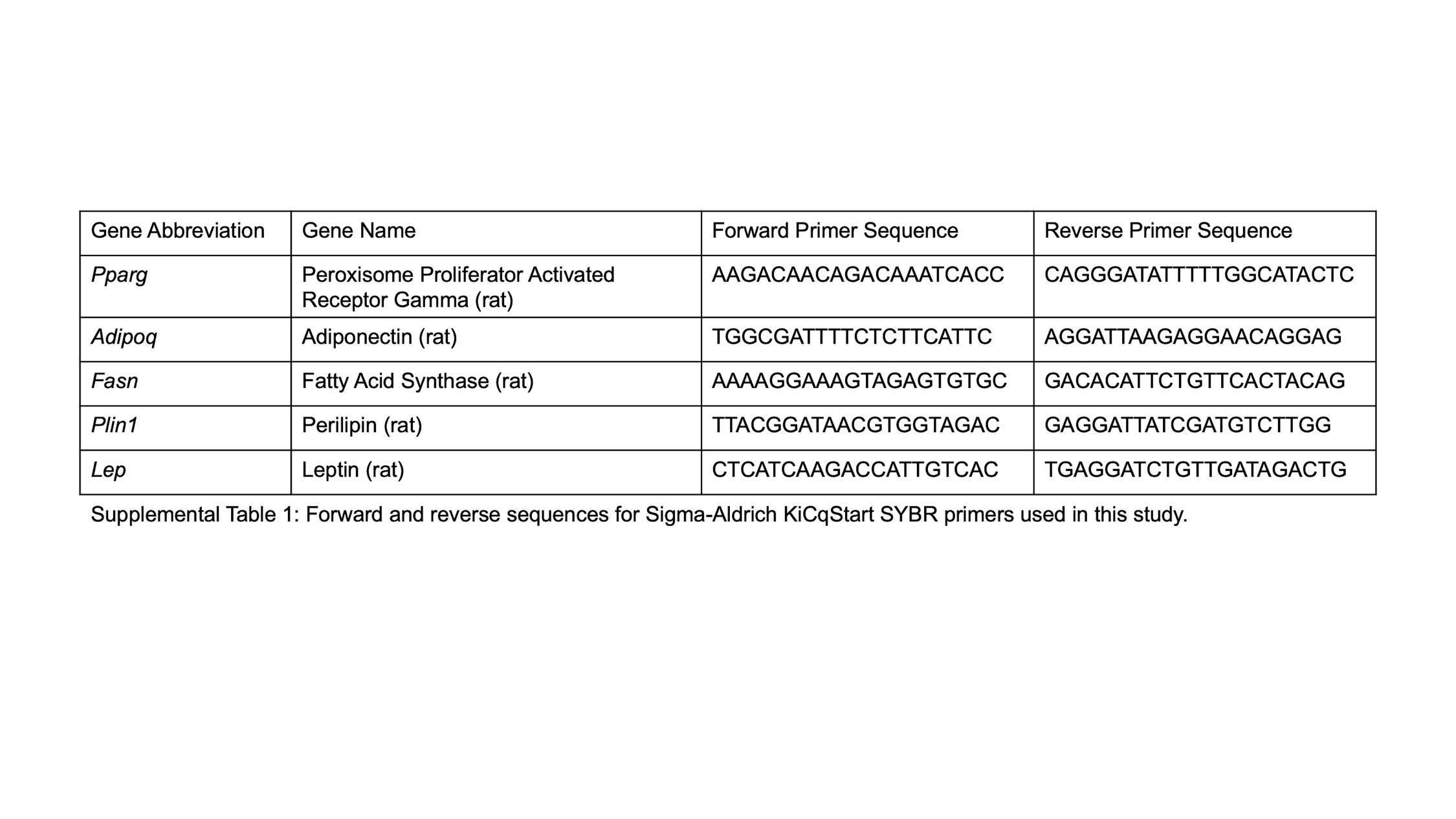

Supplement: S1 Table — (TIF) [file pone.0229251.s001.tif]

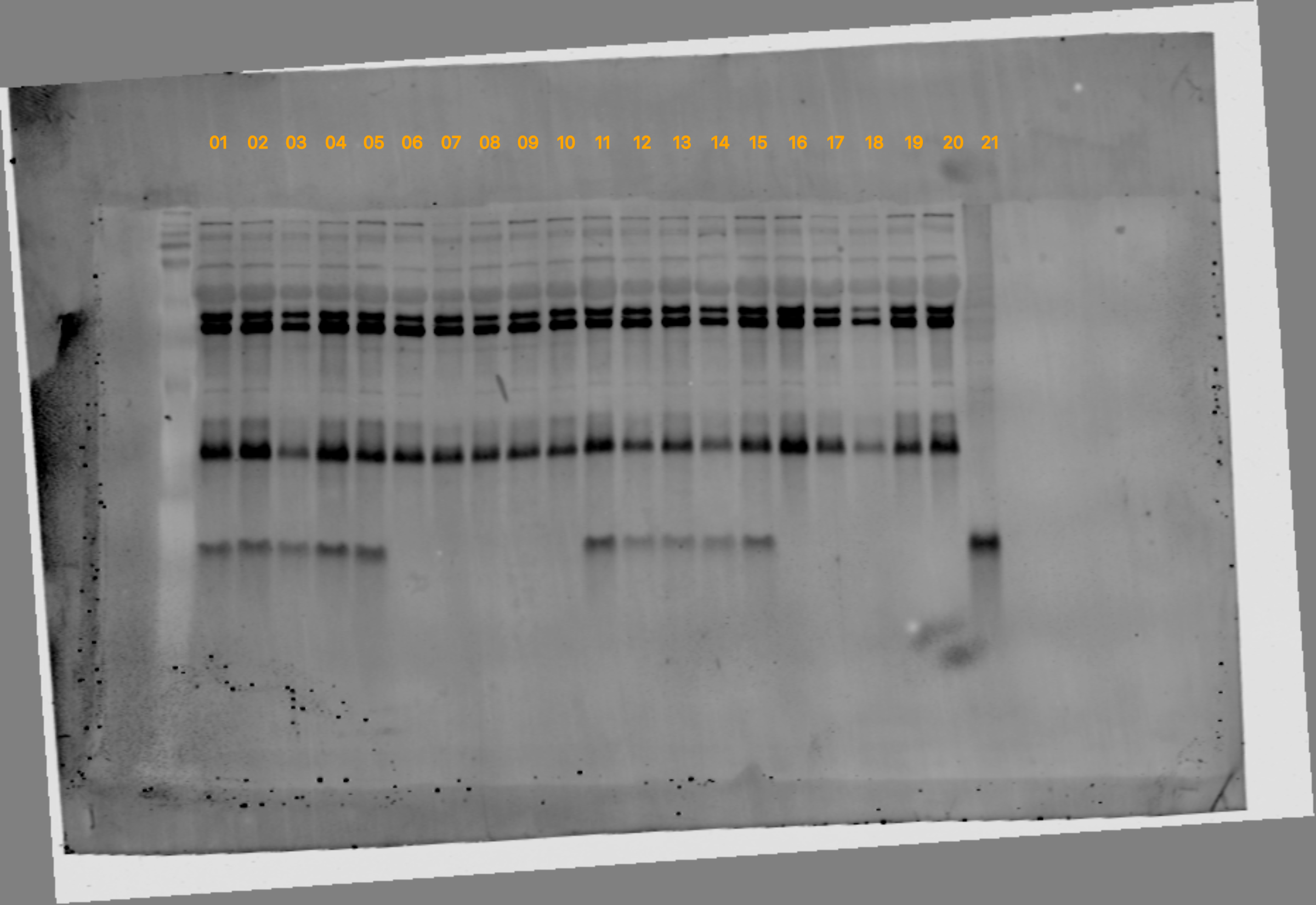

Supplement: S1 Original western — (TIF) [file pone.0229251.s002.tif]

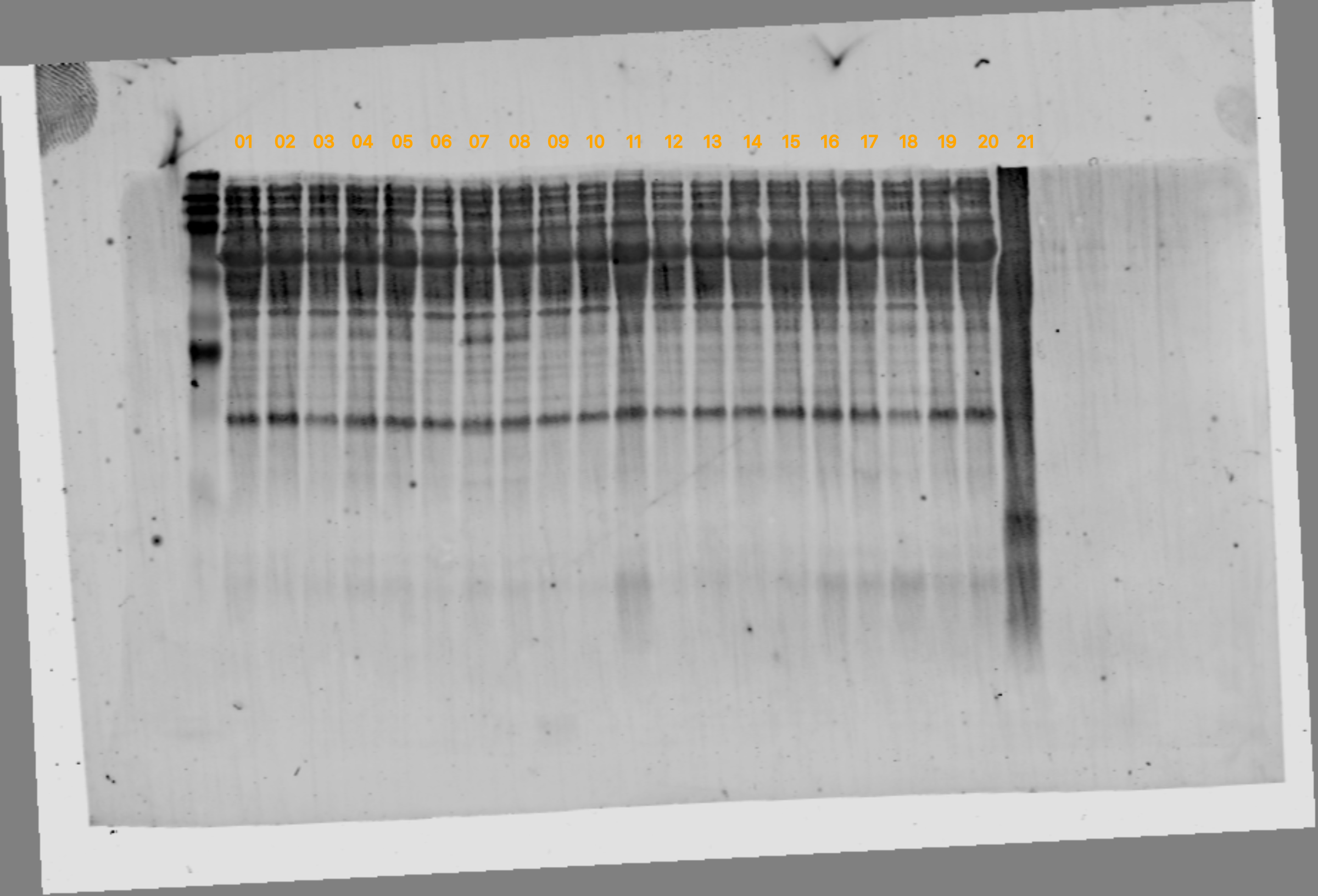

Supplement: S2 Original western — (TIF) [file pone.0229251.s003.tif]
